# A coalescence of two syndromes in a girl with terminal deletion and inverted duplication of chromosome 5

**DOI:** 10.1186/1471-2350-15-21

**Published:** 2014-02-11

**Authors:** Danijela Krgovic, Ana Blatnik, Ante Burmas, Andreja Zagorac, Nadja Kokalj Vokac

**Affiliations:** 1 Laboratory of Medical Genetics, University Clinical Centre Maribor, Ljubljanska Street 5, 2000 Maribor, Slovenia; 2 Paediatric Clinics, University Clinical Centre Maribor, Ljubljanska Street 5, 2000 Maribor, Slovenia; 3 Faculty of Medicine, University of Maribor, Slomškov trg 15, 2000 Maribor, Slovenia

**Keywords:** Deletion with inverted duplication of 5p, Cri-du-chat syndrome, Trisomy 5p, Cat-like cry, Ear agenesis

## Abstract

**Background:**

Rearrangements involving chromosome 5p often result in two syndromes, Cri-du-chat (CdC) and Trisomy 5p, caused by a deletion and duplication, respectively. The 5p15.2 has been defined as a critical region for CdC syndrome; however, genotype-phenotype studies allowed isolation of particular characteristics such as speech delay, cat-like cry and mental retardation, caused by distinct deletions of 5p. A varied clinical outcome was also observed in patients with Trisomy 5p. Duplications of 5p10-5p13.1 manifest themselves in a more severe phenotype, while trisomy of regions distal to 5p13 mainly causes mild and indistinct features. Combinations of a terminal deletion and inverted duplication of 5p are infrequent in literature. Consequences of these chromosomal rearrangements differ, depending on size of deletion and duplication in particular cases, although authors mainly describe the deletion as the cause of the observed clinical picture.

**Case presentation:**

Here we present a 5-month-old Slovenian girl, with *de novo* terminal deletion and inverted duplication of chromosome 5p. Our patient presents features of both CdC and Trisomy 5. The most prominent features observed in our patient are a cat-like cry and severe malformations of the right ear.

**Conclusion:**

The cat-like cry, characteristic of CdC syndrome, is noted in our patient despite the fact that the deletion is not fully consistent with previously defined cat-like cry critical region in this syndrome. Features like dolichocephaly, macrocephaly and ear malformations, associated with duplication of the critical region of Trisomy 5p, are also present, although this region has not been rearranged in our case. Therefore, the true meaning of the described chromosomal rearrangements is discussed.

## Background

Chromosomal rearrangements involving the short arm of chromosome 5 often result in two well-known syndromes, Cri-du-chat (CdC) and Trisomy 5p, caused by a deletion and duplication, respectively. CdC is one of the recognizable contiguous gene disorders, therefore much has been done in terms of genotype-phenotype correlations [1]. A cat-like cry is considered to be a hallmark observed in CdC patients, although clinical manifestations generally depend on the 5p segment involved in the deletion [1,2]. A distinct facial appearance with mental retardation has been delineated in these patients, associated with a deletion of 5p15.2. This region was therefore postulated to be the critical region of the syndrome [3,4]. Introducing more advanced techniques in diagnostics has allowed further determination and isolation of particular characteristics such as speech delay, cat-like cry, mental retardation, and facial dysmorphism, caused by distinct deletions of 5p in patients lacking the full clinical CdC spectrum [5,6].

A varied clinical manifestation was also observed in patients with Trisomy 5p, depending on the position of the duplication [7,8]. Duplications involving complete 5p or a small segment between 5p10-5p13.1 usually manifest in a more severe phenotype; thus, this region was proposed to be the critical region for Trisomy 5p [9-11]. Meanwhile, trisomy of regions distal to 5p13 mainly causes mild and indistinct features [12]. A combination of terminal deletion and inverted duplication of 5p is infrequent in literature. To the best of our knowledge, only six patients have been reported so far [8,13-17]. Consequences of these uncommon chromosomal rearrangements differ, depending on the size of the deletion and duplication in each particular case. This is shown in variable clinical pictures observed, although authors mainly describe deletion as causative for the clinical outcome [8,13,16,17].

Here we present a new patient with a terminal deletion and inverted duplication of chromosome 5p. Our patient presents a cat-like cry, characteristic of CdC syndrome. Features like dolichocephaly, macrocephaly and ear malformations, observed in Trisomy 5p, are also present. By comparing our patient to those previously reported, we aim to clarify the potential role of the deletion and duplication in this type of chromosomal rearrangement.

## Case presentation

### Clinical report

**Figure 1:**
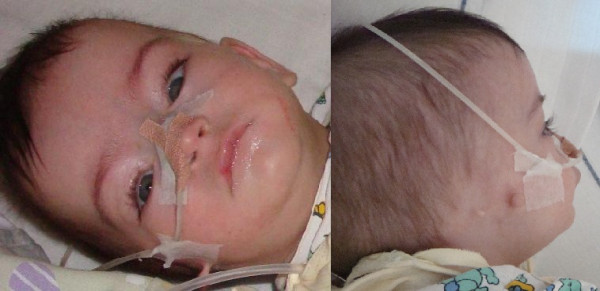
**Facial appearance of the patient at the adjusted age of 5 months.** Note dolichocephaly, relative macrocephaly, right ear agenesis with preauricular ear tags, hypertelorism and microretrognathia.

Currently 5-month-old girl was born by caesarean section at 32 weeks’ gestation due to preterm premature rupture of membranes to a 31-year old, gravida 3 para 2 mother. The pregnancy was uneventful with the exception of an episode of minor bleeding in the first trimester. The parents are healthy non-consanguineous. Two months after the delivery, the patient’s mother was diagnosed with choriocarcinoma and underwent chemotherapy and surgical treatment. The family history was otherwise unremarkable. The newborn’s birth length was 36 cm, her weight 1440 g, head circumference 28 cm. Her Apgar score was 5 at 1 minute and 6 at 5 minutes after birth due to respiratory distress and received ventilatory support for four days. Feeding became increasingly difficult at the age of two weeks, mostly due to uncoordinated movements of the supralaryngeal structures and sub sequential aspiration. The difficulties seemed to be resolving as she reached the age of two months but have reappeared since, requiring a gastrostomy tube placement. Immediately after birth, she was diagnosed with right-sided anotia with two preauricular skin tags. An MRI showed atresia of the right ear canal but normal middle and inner ear structures. Hearing tests performed on the left ear were normal. In addition, she had partial bilateral choanal atresia. An echocardiogram revealed a haemodynamically insignificant ventricular septum defect and a patent ductus arteriosus. Abdominal ultrasound examination was unremarkable, although both kidneys seemed to be smaller than expected. In the neonatal period a neurological examination revealed central hypotonia, which was less evident when she was re-examined at the adjusted age of three months. Signs of a slight developmental delay were also noted. Her electroencephalogram was normal, but a brain MRI revealed a dilated right lateral ventricle with possible periventricular heterotopia and somewhat smaller hippocampal regions. At the adjusted age of three months our patient weight was 5000 g, length 59 cm, head circumference 42.5 cm. Dysmorphic facial features were more prominent than in the neonatal period and included dolichocephaly with relative macrocephaly, right ear agenesis with preauricular ear tags, hypertelorism and microretrognathia. When she was re-examined at the adjusted age of five months, additional features were noted – convergent strabismus, long eyelashes and widely spaced nipples. Both her height and weight were at the 5th percentile for her adjusted age (Figure 1). A characteristic high-pitched cry first described directly after birth was still present.

### Methods

#### Array-CGH analysis

Array-CGH was performed on DNA extracted from peripheral blood leukocytes of the patient and her parents using the BlueGnome CytoChip ISCA 8 × 60 K platform (BlueGnome Limited, Cambridge, United Kingdom). The assay was performed according to the manufacturer’s instructions (CytoChip Oligo v1.2, 1st February 2011, BlueGnome, Cambridge, UK). The obtained data were analysed using the Blue Fuse Multi v3.1 software tool (BlueGnome, Cambridge, UK).

#### Chromosome analysis

Chromosome analysis was performed on metaphase chromosomes from peripheral blood lymphocytes on the patient and her parents. Chromosomes were harvested according to standard cytogenetic methods and analysed by G-bands.

#### FISH analysis

The deletion and duplication on chromosome 5p were verified by fluorescent *in situ* hybridisation (FISH) carried out on metaphase spreads using the following BlueFISH BAC probes: RP11-73G8 orange (5p15.32), RP11-433G24 green (5p15.31), RP11-203I22 green (5p15.31, encompassing *FLJ25076* gene), and RP11-96P21 orange (5p15.2) (BlueGnome, Cambridge, UK), according to the manufacturer’s instructions (BlueFish™ protocol v4, July 9th 2009, BlueGnome, Cambridge, UK).

### Results

#### Array-CGH analysis

**Figure 2:**
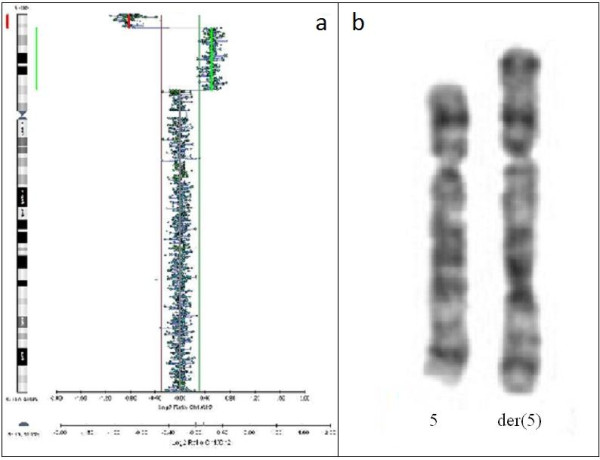
**The result of molecular and standard karyotyping of the patient. (a)** An array-CGH analysis showing a terminal deletion and duplication of 5p. The breakpoints of rearrangements were determined to be arr[hg19] 5p15.33p15.31(22,179–6,303,297) × 1 dn,5p15.31p13.2(6,414,458–36,094,217) × 3 dn. **(b)** A normal and rearranged chromosome 5 after GTG banding.

The array-CGH analysis indicated a smaller terminal deletion (6.3 Mb) and much larger interstitial duplication (29.6 Mb) on the short arm of chromosome 5. The positions of the rearrangements were determined to be arr[hg19] 5p15.33p15.31(22,179–6,303,297) × 1 dn,5p15.31p13.2(6,414,458–36,094,217) × 3 dn (Figure 2a).

#### Chromosome analysis

The chromosomal rearrangements detected by array-CGH were confirmed by karyotyping as 46,XX,der(5) (Figure 2b). The proband’s parents had normal array-CGH profiles and karyotypes (data not shown). FISH analysis was performed in order to establish the precise type of rearrangements.

#### FISH analysis

**Figure 3:**
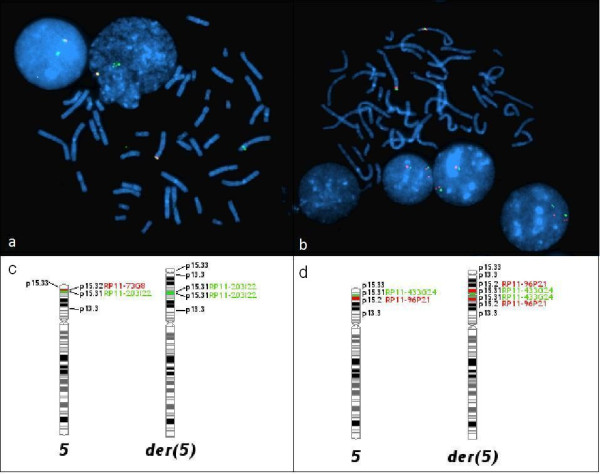
**FISH analysis. (a)** The deletion was verified using probe RP11-73G8 orange (5p15.32). A single orange signal was detected on the normal chromosome 5 and in the nucleus, whereas the signal was absent on the rearranged chromosome 5. Two green signals, one on each chromosome 5, represent the RP11-203I22 (5p15.31) probe, encompassing the *FLJ25076* gene. The brighter green signal on the rearranged chromosome 5 indicates that this gene is duplicated. **(b)** The type of duplication was determined by RP11-433G24 green (5p15.31) and RP11-96P21 orange (5p15.2) probes. Two orange signals (RP11-96P21) on each side of the green signal (RP11- 433G24) on the rearranged chromosome 5 indicated that the duplication was inverted. **(c)** An idiogram of the normal and rearranged chromosome 5 with probe positions used in Figure 3a is presented. **(d)** An idiogram of the normal and rearranged chromosome 5 with probe positions used in Figure 3b is presented.

A deletion of the 5p15.31-5pter was confirmed using a combination of the RP11-73G8 orange (5p15.32) probe in the deleted region and the RP11-203I22 green (5p15.31, encompassing *FLJ25076* gene) probe in the duplicated region. Absence of the orange signal on the rearranged chromosome 5 represents a deletion of region 5p15.32. Both probes were evident on the normal chromosome 5 (Figure 3a). The RP11-203I22 (5p15.31) probe also covers the whole *FLJ25076* gene. From the brighter and larger green signal observed on the rearranged chromosome 5, we concluded that the gene was duplicated in our case (Figure 3a). The inverted duplication was confirmed using RP11-433G24 green (5p15.31) probe, which lies distal to the breakpoint of two described rearrangements and RP11-96P21 orange (5p15.2) probe, which lies within the critical region of CdC syndrome. Two orange signals (RP11-96P21) on either side of a green signal (RP11-433G24) on the rearranged chromosome 5 represented an inverted duplication of the 5p13.2-5p15.31 (Figure 3b). According to all methods used, the proband’s karyotype could be written as 46,XX,der(5).ish der(5)del(5)(p15.31p15.33)inv dup(5)(p13.2p15.3)(RP11-73G8-,RP11-203I22++,RP11-96P21++,RP11-433G24+).arr[hg19] 5p15.31p13.2(6414458–36094217) × 3 dn,5p15.33p15.31(22179–6303297) × 1 dn.

### Discussion

Although array-CGH analysis has become a widely used method in diagnostics of children with developmental delays, intellectual disabilities and congenital anomalies, incidence of complex rearrangements involving chromosome 5p as described here is still very low. Vetro et al. [14] assumed that these rearrangements are underestimated due to the limitations of the standard cytogenetic techniques used, and methods like array-CGH will enable new cases to be discovered. Only three new cases have been reported since the Vetro et al. [14] study and six altogether [8,13-17], reflecting the rarity of this phenomenon.

In 1999, Sreekantaiah et al. [8] described the first case of a terminal deletion with inverted duplication of 5p in a 4-year-old girl with developmental delay and cat-like cry noted at birth. Minor dysmorphic features were also observed, but the subject lacked other characteristics of CdC syndrome. In subsequent years, five more cases have been reported, two postnatal and three prenatal. Wang et al. [13] reported a 6-year-old boy with speech and motor skill delay, without CdC features. Vetro et al. [14] presented a prenatal case with cystic hygroma and other congenital facial and body anomalies, including dolichocephalia, low-set ears with malformed helices, microretrognathia and others. A newborn with similar chromosomal rearrangements of 5p was described by Vera-Carbonell et al. [15], presenting both features of CdC syndrome and Trisomy 5p. The patient died at the age of three months due to a congenital heart defect. Recently, two more prenatal cases were described by Mosca et al. [16] and Izzo et al. [17]. The first presented a deformed skull, facial asymmetry, hypertelorism, exophthalmia, microretrognathia, long philtrum and large mouth, short nasal ridge, anteverted nostrils, bilateral rocker-bottom feet, and hypoplastic helix [16]. A patient from Izzo et al. [17] displayed only mild phenotypic abnormalities and facial dysmorphisms. Here we present a new case of *de novo* terminal deletion with an inverted duplication in a 5-month-old girl. Our patient presents features of both CdC and Trisomy 5. The most prominent features observed in our patient are a cat-like cry and severe malformations of the right ear.

All above mentioned cases describe terminal deletions followed by inverted duplications of varied size. When comparing phenotypes, we see clear differences between patients, including our own (Table 1). The hypotonia, psychomotorical retardation or developmental delay, hypertelorism, microretrognathia, and low-set ears observed in our proband are characteristics shared by both CdC syndrome and Trisomy 5p [1,18]. The first two listed features and the cat-like cry, a hallmark of CdC, are present in three out of four postnatal patients. Additionally, strabismus, feeding problems, and respiratory difficulties described in Trisomy 5p are present in two out of four live births. Hypertelorism was observed in three, microretrognathia and low-set ears in four out of seven patients. Dolichocephaly and macrocephaly, features involved in Trisomy 5p, are described in two and one patient, respectively. Specific features noted in our patient are right-sided anotia with atresia of the right ear and choanal atresia. Although ear malformations are present in Trisomy 5p [7], severe ear anomalies are not frequently reported. Minor ear malformations are observed in two other patients with a deletion and inverted duplication of 5p. The preauricular skin tags seen in our patient and the patient described by Wang et al. [13] are occasionally described in CdC patients [1].

**Table 1:** CdC and Trisomy 5p features noted in patients with terminal deletion and inverted duplication of 5p

	***Sreekantaiah *** **et al. *1999 *** [8]	***Wang *** **et al. *2008 *** [13]	***Vetro *** **et al. *2008 *** [14]	***Vera-Carbonell *** **et al. *2009 *** [15]	***Mosca *** **et al. *2011 *** [16]	***Izzo *** **et al. *2012 *** [17]	***Our patient 2013***	
*Deletion*	5p15.33p15.3	5p15.33p15.31	5p15.33p14.1	5p15.33p14.2	5p15.33p15.3	5p15.33	5p15.33p15.31	*Deletion*
*Size*	9.9 Mb	6.9 Mb	25 Mb	24.34 Mb	6.6 Mb	870 kb	6.3 Mb	*Size*
*Duplication/inversion*	5p15.3p14	5p15.31p14.3	5p14.1p11	5p14.2p13.1 with duplication to 5p12	5p15.31	5p15.33p13.1	5p15.31p13.2	*Duplication/inversion*
*Size*	10 Mb	13 Mb	20.3 Mb	20.72 Mb	2.3 Mb	40.5 Mb	29.7 Mb	*Size*
*Heredity*	*De novo*	*De novo*	*De novo*	*De novo*	*De novo*	*de novo*	*De novo*	*Heredity*
*Age of the patient*	4 years	6 years	prenatal	Newborn	Prenatal	Prenatal	Newborn	*Age of the patient*
*Gender*	F	M	M	F	M		F	*Gender*
*Pregnancy/Birth*	Normal	Premature	Termination	39 weeks	Termination	Termination	Premature	*Pregnancy/Birth*
*Trisomy 5p features*								*Cri-du-chat features*
*Head*								*Head*
*Dolichocephaly*			+				+	
*Macrocephaly*							+	
*Hypertelorism*			hypo-	+	+		+	*Hypertelorism*
*Microretrognathia*			+	+	+		+	*Microretrognathia*
*Ears*								*Ears*
*Low-set ears*			+	+		+	+	*Low-set ears*
		+					+	*Preauricular skin tags*
*Dysplastic ears*			+		+ (left)		+ (left)	
*Nose*								*Nose*
*Broad nasal bridge*				+			+	*Broad nasal bridge*
*Eyes*								*Eyes*
*Epicanthal folds*							+	*Epicanthal folds*
*Strabismus*	+						+	
*Heart*								*Heart*
*CHD*			+	+			+	*CHD*
*Neurological*								*Neurological*
*Developmental delay*	+	+					+	*Developmental delay*
*Hypotonia*		+		+			+	*Hypotonia*
*Cry*								*Cry*
	+			+			+	*High-pitched cry*
*Others*								*Others*
*Feeding problems*				+			+	
*Failure to thrive*		+		+			+	

A genotype-phenotype correlation of CdC syndrome has been carried out in the past, in order to distinguish the critical genomic regions for the cat-like cry, distinct facial dysmorphism, microcephaly, and severe psychomotor delay and mental retardation, features most prominent in the first year of life in CdC patients [1]. Several of these studies mapped the critical regions to different positions on the 5p, leading to conflicting results [2,3,19,20]. Two distinct cat-like cry critical regions have been mapped, one to the proximal part of 5p15.3 [3,19,20] and one to the distal part of 5p15.2 [2]. The array-CGH analysis allowed more precise localization of the cat-like cry critical region between 6.0–7.5 Mb on 5p15.31 [6]. The region was further narrowed in the study by Wu et al. [5], using quantitative PCR, to a 640 kb small region on 5p15.31, containing just three candidate genes *FLJ25076*, *FLJ20303*, and *MGC5309*. Among these three genes, gene *FLJ25076* was proposed as a candidate gene. Namely, expression profiles determined by quantitative PCR showed that the *FLJ25076* gene is locally expressed in thoracic and scalp tissue, whereas the other two genes are expressed uniformly in all tissues tested [5]. Interestingly, the genes proposed by this study are duplicated in our case. Duplication has been confirmed with both array-CGH and FISH analysis (Figures 2a and 3b). We used the RP11-203I22 green (5p15.31) probe, specific to the *FLJ25076* gene, most plausible of the proposed genes. Two green signals on chromosomes 5 confirmed the presence of this gene on both the normal and the rearranged chromosome 5. A bright green patch signal on the rearranged chromosome 5 and its position indicate that the *FLJ25076* gene is duplicated in this chromosome (Figure 3a).

An almost 30 Mb large duplication identified in our patient, which resides on 5p13.2-5p15.31, lies just outside the critical region 5p10-5p13.1 postulated for Trisomy 5p syndrome [9-11,15]. However, vague boundaries were also reported in this syndrome, putting the critical region between 5p10-5p13.3 [21-23]. Nevertheless, it is generally accepted that duplications of regions located proximal to 5p13 most likely have greater significance in the clinical severity in Trisomy 5p than duplications involving regions distal to 5p13. Dolichocephaly, macrocephaly, microretrognathia, low-set dysplastic ears, congenital heart, and respiratory defects noted in our proband are only some of the features described in the Trisomy 5p phenotype. Some of these features are also shared with features of CdC. Low-set ears and microretrognathia are noted in most CdC patients, whereas cardiac anomalies and respiratory infections are not frequently reported [1]. Therefore, according to the phenotype described in patients with Trisomy 5p, we conclude that dolichocephaly and macrocephaly are features caused by the duplication determined in our proband. A right-sided anotia with atresia of the right ear is also consistent with dysplastic ears reported in 5p duplication patients [21], although the described patients usually present dysplastic ears in terms of malformed helix and not severe ear malformation as found in our proband.

Intertwining phenotypic features of both CdC and Trisomy 5p syndromes in patients with a terminal deletion with inverted duplication make it difficult to clearly distinguish the effect of the individual chromosomal rearrangements on the clinical outcome. Based on the position of the deletion, which in our case lies between 5p15.31-5pter, we deduce that the cat-like cry, a hallmark of CdC syndrome, originates from the above-mentioned deletion of the region. Where exactly the critical region is located remains uncertain. As mentioned above, the *FLJ25076*, *FLJ20303*, and *MGC5309* genes postulated in the study by Wu et al. [5] are duplicated in our case, which is inconsistent with their findings. Interestingly, Wang et al. [13] reported a patient with a 6.9 Mb terminal deletion followed by an inverted duplication, who did not present a cat-like cry at birth. They suggested that the critical region for the cat-like cry resides within the 0.6 Mb region between 6.9–7.5 Mb, which also conflicts with our findings. According to the deletion described in our proband and the cat-like cry region (deletion between 6.0–7.5 Mb) proposed by Zhang et al. [6] we can assume that the genes responsible for this feature are located in the region between 6.0–6.3 Mb on 5p15.31. Unfortunately, positioning of the cat-like cry critical region and isolation of the candidate genes is not feasible at this moment in time, suggesting a higher need for more precise genotype-phenotype studies. Nevertheless, the uncoordinated movements of the supralaryngeal structures noted in our proband are, apart from feeding difficulties, probably also connected to the cat-like cry. Our patient’s deletion also encompasses a critical region for speech delay in CdC syndrome associated with 3.2–6.4 Mb sized rearrangement of the 5p15.3 band [2,6]. Currently, no information regarding this feature can be provided for our patient, but it is possible that she will have difficulties with speech in the future.

In our case, the impact of the duplication should be stressed. Contrary to the earlier publications of terminal deletions with inverted duplications, where minor impact of duplication has been suggested [8,13,16,17], the duplication in our case seems to be crucial for the observed clinical picture. Some features, such as macrocephaly, dolichocephaly, and dysplastic ears may not be induced by the duplication of a critical region of Trisomy 5p, and are a result of the duplication of genes positioned distal to 5p13. It is also possible that the Trisomy 5p critical region does not extend to the whole 5p13 band as Izzo et al. [17] proposed previously. Interestingly, although the duplication reported in their case exceeds the size of the duplication reported in this paper, a milder phenotype was observed in their patient. This only confirms the complexity of the Trisomy 5p phenotype. Newly described patients with duplications in this region will enable more precise limitation of the mentioned region and clarify genotype-phenotype correlations.

## Conclusion

To conclude, this report indicates that the actual state of the complex phenotype observed in our patient is likely due to the influence of both chromosomal aberrations and not primarily due to the deletion, as described previously in patients with a terminal deletion with inverted duplication. Namely, the most prominent phenotypic features noted in our patient, except from the cat-like cry, are results of the chromosome 5p duplication. Also, the patient follow-up will contribute to better understanding of the impact of the 5p deletion on speech delay. Comparison of our patient with new CdC patients with a cat-like cry will hopefully contribute to narrowing the critical region for this feature, while the described duplication could be conducive to more accurate genotype-phenotype correlations in Trisomy 5p in future.

## Consent

Written informed consent was obtained from the patient’s parents to take part in the study as well as for publication of the images (including full-face pictures). A copy of the written consent is available for review by the Editor-in-Chief of this journal.

## Abbreviations

CdC: Cri-du-chat; MRI: Magnetic resonance imaging; CGH: Comparative genomic hybridization; FISH: Fluorescent *in situ* hybridisation; BAC: Bacterial artificial chromosome; PCR: Polymerase chain reaction; Mb: Mega base; CHD: Congenital heart defects.

## Competing interests

The authors declare that they have no competing interests.

## Authors’ contributions

DK wrote the manuscript and carried out the molecular karyotyping and data analysis; AB and AB performed a clinical analysis of the patient; AZ conducted the cytogenetic analysis; and NKV coordinated the study. All the authors have read and approved the manuscript.

## Pre-publication history

The pre-publication history for this paper can be accessed here:

http://www.biomedcentral.com/1471-2350/15/21/prepub
